# Lessons from a theory of change-driven evaluation of a global mental health funding portfolio

**DOI:** 10.1186/s13033-021-00442-6

**Published:** 2021-02-27

**Authors:** G. Miguel Esponda, G. K. Ryan, G. Lockwood Estrin, S. Usmani, L. Lee, J. Murphy, O. Qureshi, T. Endale, M. Regan, J. Eaton, M. De Silva

**Affiliations:** 1 Health Service and Population Research Department, Institute of Psychiatry, Psychology and Neuroscience, King’s College London, 16 De Crespigny Park, Camberwell, London, SE5 8AB UK; 2 ESRC Centre for Society and Mental Health, King’s College London, London, UK; 3 Department of Population Health, London School of Hygiene and Tropical Medicine, London, UK; 4 Centre for Brain and Cognitive Development, Department of Psychological Sciences, Birkbeck College, University of London, London, UK; 5 Independent Researcher, Minneapolis, MN USA; 6 Independent Researcher, London, UK; 7 Department of Psychiatry, Faculty of Medicine, University of British Columbia, Vancouver, BC Canada; 8 Department of Counselling and Clinical Psychology, Teachers College, Columbia University, New York, NY USA; 9 Health Improvement Directorate, Public Health England, London, UK; 10 CBM Global, Cambridge, UK; 11 Department of Population Health, Wellcome Trust, London, UK

**Keywords:** Global mental health, Theory of change, Implementation

## Abstract

**Background:**

Given the underinvestment in global mental health to-date, it is important to consider how best to maximize the impact of existing investments. Theory of Change (ToC) is increasingly attracting the interest of funders seeking to evaluate their own impact. This is one of four papers investigating Grand Challenges Canada’s (GCC’s) first global mental health research funding portfolio (2012–2016) using a ToC-driven approach.

**Methods:**

A portfolio-level ToC map was developed through a collaborative process involving GCC grantees and other key stakeholders. Proposed ToC indicators were harmonised with GCC’s pre-existing Results-based Management and Accountability Framework to produce a “Core Metrics Framework” of 23 indicators linked to 17 outcomes of the ToC map. For each indicator relevant to their project, the grantee was asked to set a target prior to the start of implementation, then report results at six-month intervals. We used the latest available dataset from all 56 projects in GCC’s global mental health funding portfolio to produce a descriptive analysis of projects’ characteristics and outcomes related to delivery.

**Results:**

12,999 people were trained to provide services, the majority of whom were lay or other non-specialist health workers. Most projects exceeded their training targets for capacity-building, except for those training lay health workers. Of the 321,933 people screened by GCC-funded projects, 162,915 received treatment. Most projects focused on more than one disorder and exceeded all their targets for screening, diagnosis and treatment. Fewer people than intended were screened for common mental disorders and epilepsy (60% and 54%, respectively), but many more were diagnosed and treated than originally proposed (148% and 174%, respectively). In contrast, the three projects that focused on perinatal depression exceeded screening and diagnosis targets, but only treated 43% of their intended target.

**Conclusions:**

Under- or over-achievement of targets may reflect operational challenges such as high staff turnover, or challenges in setting appropriate targets, for example due to insufficient epidemiological evidence. Differences in delivery outcomes when disaggregated by disorder suggest that these challenges are not universal. We caution implementers, funders and evaluators from taking a one-size-fits all approach and make several recommendations for how to facilitate more in-depth, multi-method evaluation of impact using portfolio-level ToC.

## Background

### Investing in global mental health

Despite growing recognition of the importance of mental health to political and development agendas [1], median government expenditure on mental health ranges from just 0.02 United States dollars (USD) per capita in low-income countries to 2.62 USD in upper middle-income countries [2]. In sub-Saharan Africa, for example, this amounts to less than 1% of countries’ overall health budgets. Meanwhile, only 0.4% of all overseas development assistance for health is allocated to mental health [3]. This is in stark contrast to the high prevalence of mental, neurological and substance use (MNS) disorders, which may contribute up to 13.03% of the global burden of disease [4].

Redressing these imbalances by increasing local and international investment in mental health has been a key priority for the global mental health movement since its inception [5]. Research efforts have focused on garnering evidence for investment, for example by demonstrating the cost-effectiveness of mental health interventions that increase access to care in low-resource settings. Many interventions have proven successful in improving health and functional outcomes, and have even garnered international attention in the media [6] and at high-profile events for policy-makers [7, 8] and other funders [9]. Yet investment in mental health remains stubbornly low, even when compared to other health sectors. To illustrate: from 2010–2016 nearly half of all disability assistance for health was spent on the control of sexually-transmitted diseases such as HIV/AIDS, while HIV/AIDS was responsible for less than 5% of the global burden of disease [10].

Given the relative underinvestment in global mental health to-date, it is important to consider how best to maximize the impact of existing investments, and funders often seek evidence of value for money to support further funding decisions. Numerous priority-setting exercises have been undertaken to ensure that the limited resources available for mental health in low- and middle-income countries (LMICs) are used efficiently to target strategic issues, particularly in terms of research [5, 11–14]. Less attention has been paid to ensuring that funding made available for global mental health is used to maximum effect. Theory of Change (ToC), which in recent years has become a popular tool for the design and evaluation of complex interventions in global mental health [15], is increasingly attracting the interest of funders seeking to evaluate their own impact [16–18]. This is one of four papers investigating Grand Challenges Canada’s (GCC’s) first global mental health research funding portfolio, using a ToC-driven approach.

### Evaluating a global mental health funding portfolio

Launched in 2010, GCC is a non-profit organisation funded by the Canadian Government and other partners. It is one of relatively few development organisations that has invested in a funding programme dedicated to global mental health. By 2016, GCC had committed $28,232,030 CAD to 51 projects in its Global Mental Health Programme and leveraged an additional $1,297,946 CAD in co-funding [19]. Funding was also made available to mental health projects via the GCC Stars in Global Health and Transition to Scale programmes (launched in 2010 and 2013, respectively). Together, these programmes created a pipeline granting innovators seed funding to demonstrate proof of concept, with the potential for further funding to support larger-scale intervention and implementation research.

This pipeline structure is consistent with GCC’s commitment to “an evidence-based approach to development innovation” (Grand Challenges Canada, n.d.) and the nature of innovation seed funding more broadly, where potential for scale and wider population benefit are core to the investment model. Grantees must evidence the viability and transformative potential of their innovation at each stage of the pipeline before moving on to the next. Further, as a government-funded organisation, GCC must maintain transparency by demonstrating how taxpayer dollars have been used and to what ends. Consequently, GCC requires a high level of routine reporting from its grantees, collating process and outcome data across its Results-based Management and Accountability Framework (RMAF) and evaluating the success of its various funding portfolios in terms of ‘number of lives improved’ [19].

The result is a wealth of data available from grantees’ earliest stages of seed funding— and in some cases through to scale-up—for a diverse portfolio of mental health projects carried out over a similar timespan via a single funder. This provides a unique opportunity for research and evaluation. For example, overarching questions related to human resources, case detection and accessibility of health care services can be examined across different projects that share these common intervention elements, providing a broad view of many of the key issues and practical challenges in improving mental health in LMIC settings.

In this paper, we share descriptive results of a quantitative analysis of the GCC portfolio’s Core Metrics data, and examine the strengths and limitations of a ToC-driven approach to portfolio-level evaluation, for which there is very little practical guidance currently available [16]. Related papers by Endale et al. [21], Murphy et al. [22] and Qureshi et al. [23] in this series describe the qualitative components of this evaluation, with a focus on barriers and facilitators to successful implementation across three key areas: (i) stakeholder engagement; (ii) capacity building; and (iii) service delivery. Our aim is to harness and share learning from one of the biggest investments in global mental health to-date, relevant both to funders like GCC and to implementers working in the field.

## Methods

We carried out a multi-method, ToC-driven evaluation of GCC’s 2012–2016 global mental health investment portfolio. This portfolio consists of 56 mental health projects funded through the Global Mental Health and Transition to Scale programmes. Our objectives were:

1. To describe the characteristics of the mental health projects included in the GCC portfolio.
2. To assess the extent to which grantees achieved their pre-identified outcomes on a collective pathway of change.
3. To illustrate the use of a multi-method ToC-driven methodology as a means of synthesising key data and learning regarding the implementation of a diverse portfolio of projects.
4. To investigate, using qualitative methods, factors affecting implementation that may help or hinder progress along the pathway of change (reported elsewhere in this series).

### Evaluation framework

#### Portfolio-level theory of change

From 2013 to 2016, GCC funded an innovator support platform called the Mental Health Innovation Network (MHIN, www.mhinnovation.net). MHIN is a collaboration between the Centre for Global Mental Health at the London School of Hygiene and Tropical Medicine (LSHTM) and the Department of Mental Health and Substance Abuse at the World Health Organization (WHO). One of the key services that MHIN provided to GCC was assistance in portfolio-level monitoring and evaluation.

To evaluate the collective impact of mental health projects across GCC’s funding programmes, researchers at MHIN proposed a ToC-driven approach. ToC is “a theory of how and why an initiative works”, laying bare the causal pathway by which an initiative aims to achieve impact [18]. Often a ToC is depicted visually as a diagram and developed in consultation with key stakeholders, with additional benefits for consensus-building and communication [15, 24]. By assigning indicators to short-, medium- and long-term outcomes along the causal pathway, a ToC can be especially valuable as an evaluation framework. Pin-pointing where on this causal pathway an initiative fails to produce expected outcomes can help to “unpack the black box” of evaluation [25], distinguishing between “ideas that don’t work” (theory failure) and “ideas that haven’t been properly tested” (implementation failure) [26].

At the time of GCC’s Global Mental Health Programme launch, ToC was already in use by international mental health research consortia like PRIME (PRogramme for Improving Mental hEalth care) [27]. Utilizing a combination of country-specific and overarching, cross-country ToC maps, PRIME demonstrated that it is possible to simultaneously monitor and evaluate necessarily heterogenous, complex interventions both at the individual country level and collectively across participating country sites. PRIME’s Nepal site has also demonstrated that ToC can be used in combination with methods of qualitative comparative analysis (QCA) to identify which conditions are necessary and sufficient to bring about change [28].

In international development more broadly, ToC is increasingly being used by funders to plan, monitor and evaluate their portfolios [17, 18, 25, 29]. A ToC can be empirically tested and amended iteratively to reflect new learning, providing a road-map for current and future investment [18, 25, 29, 30]. Funders may be encouraged to develop an a priori ToC map before starting the selection process, to gauge how each potential applicant can contribute to the envisioned pathway of change [17, 25]. However, particularly in relatively young fields such as global mental health, grantees may have more specialist expertise and experience than their funders [17, 25]. Under these circumstances, grantees can play an important role in defining the pathway by which they expect the funding they receive to help achieve the funders’ desired impact [17, 25]. In the case of GCC, a ToC was developed through an iterative process involving grantees and representatives of the funding organisation, facilitated by experienced evaluators from the MHIN team at LSHTM.

#### Development of the theory of change

An initial ToC workshop was held at a Grand Challenges Community Meeting in Rio de Janeiro, Brazil, in October 2013. A working statement of impact was agreed and outcomes were backward-mapped onto a ToC diagram (Appendix 1). Indicators were suggested for each outcome under a proposed ceiling of accountability, defined by De Silva et al. (2014, p. 5) as the level at which you “stop accepting responsibility for achieving those outcomes… often drawn between the impact and the long term outcome”. Because only a very small proportion of GCC grantees would go on to receive funds for “Transition to Scale”, this ceiling of accountability was drawn under “Scale-Up”.

Grantees also received training and elective one-to-one support to develop their own project-specific ToC diagrams. These were compared to the portfolio-level ToC, which was then revised accordingly. Further adjustments were made upon review of grantees’ ‘Core Metrics’ reporting (described below), again to ensure that the portfolio-level ToC adequately reflected the component projects. Revisions to the portfolio-level ToC were presented to grantees and representatives of the funding organisation during annual GCC meetings, for feedback. Final changes were made in April 2015, at which point the ToC was “locked” for evaluation (i.e., outcomes and indicators could no longer be changed without disrupting data collection, as described further below).

### Data collection

As GCC already had a mandatory RMAF reporting system in place, proposed portfolio-level ToC indicators were adjusted where possible to align with existing indicators. The goal was to minimise the burden of reporting placed on grantees, while still collecting data against essential process and outcome indicators for monitoring and evaluation of the overall portfolio. The result was a Core Metrics Framework introduced in 2015, consisting of 23 indicators linked to the 17 outcomes of the ToC map (Appendix 1). These outcomes were grouped under four domains: project development (n = 4), delivery (n = 6), evaluation (n = 3), and context (n = 4). For each indicator relevant to their project, the grantee was asked to set a target prior to the start of implementation, then report results (for example the number of people they expected to treat through the project). Reports were submitted by grantees to GCC and transferred to MHIN at six-month intervals for analysis.

### Analysis

The quantitative analysis was conducted between November 2016 and March 2017. We used the latest available data from all GCC projects (n = 56) related to delivery outcomes (Table 1). We used descriptive statistics to describe the projects’ characteristics, results based on indicators and to compare projects’ level of achievement in relation to their intended targets. All analyses were conducted using IBM SPSS Statistics (Version 25).

**Table 1:** Summary of indicators for delivery outcomes

Outcome	Indicator
1. Adequate ongoing management, supervision and quality improvement procedures in place	Continuous quality improvement (CQI) mechanism in place (e.g. regular supervision, repeat training, other CQI methods)
2. Number of service providers (intermediaries) trained	Numerator: Number of service providers (intermediaries) trained Denominator: Target number of service providers (intermediaries) to be trained
3. Target population (beneficiaries) with mental health disorders identified	Numerator: Number of people in target population (beneficiaries) screened and identified Denominator: Target number of people to be screened and identified
4. Health promotion innovations are accessible	Numerator: Proportion of target population with access to innovation medium (e.g. television, radio, internet) Denominator: Expected proportion of target population with access to the innovation medium (e.g. television, radio, internet)
5. Target population (beneficiaries) receive integrated innovation as intended	Numerator: Number of people (beneficiaries) who received innovation (disaggregated by diagnosis, level of care, year of project etc.) Denominator: Target number of people to receive innovation

Our analysis plan was affected by several data limitations, despite numerous efforts to contact grantees both directly and via GCC to verify project data. First, large amounts of data were missing, mainly because not all outcomes were applicable to all projects. However, it was not always possible to differentiate between data that were missing due to irrelevance and data that were missing due to purposeful or accidental omission or inadequate monitoring and evaluation. Missing data was a particularly big issue for the project development outcomes. Second, given that many grantees did not report data on the outcomes of service users or other beneficiaries (e.g. family members) within the GCC-funded timeframe, most of the data collected against the Core Metrics Framework were related to implementation. Some grantees never completed their evaluations of beneficiaries’ outcomes, and others were protective of their results during the long embargo period for publication in peer-reviewed journals. Third, our quantitative approach was ill-fitted to some of the more heterogeneous outcomes that proved difficult to categorise (e.g. outcomes related to context) and were better described through rich qualitative descriptions. Due to these challenges, we limited our quantitative analysis to delivery outcomes (Table 1).

In the analysis of delivery data, there were several instances when disaggregated data was not provided by grantees (e.g. for types of providers trained or types of diagnoses screened, diagnosed and treated). Regarding types of diagnoses, several projects targeted more than one disorder therefore in the absence of disaggregated data it was impossible to know the number of people that had been screened, diagnosed or treated in each category. We only present disaggregated data when available and report the number for which disaggregated data is not available.

## Results

### Project characteristics

The global mental health investment portfolio consisted of 56 projects from the Global Mental Health and Transition to Scale programmes. The characteristics of these projects and the subsample that participated in the qualitative component are summarised in Table 2. Thirty-five projects (62%) targeted more than one disorder, life stage, population group and/or project component. Common mental disorders were the most frequently targeted (52%), followed by behavioural and emotional disorders (39%). The number of projects targeting adults (37%) was similar to the number targeting children and young adolescents (41%) and women (39%). The highest proportion of projects were located in Africa (45%), followed by South Asia (27%). Most projects carried out capacity building activities (95%), treatment, care and rehabilitation (88%), and stakeholder engagement (79%).

**Table 2:** General characteristics of included GCC mental health projects

	Core Metrics analysis (n = 56) N (% of total)	Qualitative study (n = 29) N (%)
Target disorder		
Common mental disorders	29 (52)	16 (55)
Behavioural and emotional disorders	22 (39)	13 (45)
Epilepsy and seizures	15 (27)	5 (17)
Severe mental disorders	12 (21)	6 (21)
Trauma and PTSD	12 (21)	7 (24)
Suicide and self-harm	11 (20)	7 (24)
Developmental disorders	10 (18)	7 (24)
Alcohol and substance use disorders	9 (16)	5 (17)
Dementia	3 (5)	2 (7)
All	6 (11)	3 (10)
Target life stage		
Newborns	5 (9)	2 (7)
Infants, children and early adolescents	23 (41)	14 (48)
Adults (including young adults)	21 (37)	13 (45)
Elderly	9 (16)	6 (21)
Target population		
Women	22 (39)	9 (31)
Vulnerable groups (e.g. conflict affected populations)	16 (29)	10 (34)
General population (any life stage)	12 (21)	12 (41)
Region		
Africa	25 (45)	10 (35)
South Asia	15 (27)	11 (38)
Central America and the Caribbean	9 (16)	4 (14)
South East Asia	9 (16)	3 (10)
South America	6 (11)	1 (3)
Project components		
Capacity building	54 (95)	23 (79)
Detection, treatment, care and rehabilitation	49 (88)	22 (76)
Stakeholder engagement	44 (79)	12 (41)
Promotion and awareness	22 (39)	18 (62)

### Capacity building (outcomes 1 and 2)

**Fig. 1:**
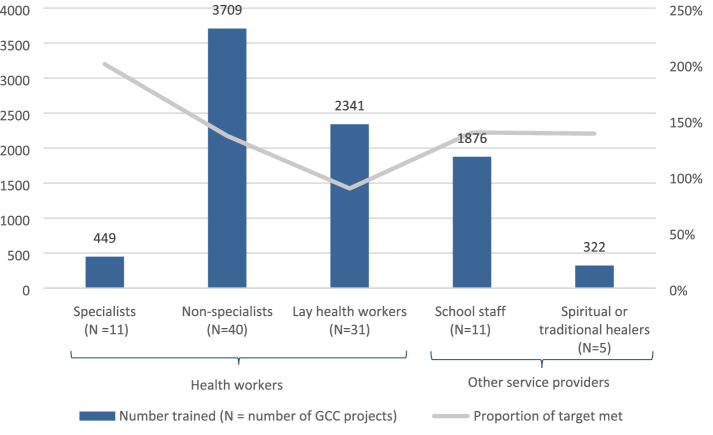
Number of providers trained in each cadre. *Missing disaggregated data for 4,302 people who received training

Capacity building activities included the delivery of training (n = 54) and use of quality assurance mechanisms (n = 49) (Table 3). Most training activities were fully or partially delivered by specialists through multiple face-to-face sessions. Two projects did not report the number of people they trained; however, the remaining 52 trained a total of 12,999 people, the majority of whom were lay workers and other non-specialist health workers [Fig. 1]. The number of participants in quality assurance activities was not systematically reported. Supervision was the most frequently used quality assurance mechanism, which in most cases was delivered on a weekly basis and by specialists or project staff.

**Table 3:** General characteristics of training and quality assurance mechanisms used by GCC projects

Type of training	GCC projects (n = 54) N (%)
Face-to-face	38 (70)
Online	2 (4)
Face-to-face and online	14 (26)
Frequency of training	
Multiple sessions (range 2–10 sessions)	50 (92)
One session	4 (8)
Training provider	
Specialists	35 (65)
Non-specialists	2 (4)
Specialists and non-specialists	8 (15)

Type of quality assurance mechanism	GCC projects (n = 49) N (%)
Supervision	46 (94)
Refresher training	20 (41)
Information system	8 (16)
Evaluation	7 (14)
Programme manager	6 (12)
Frequency of contact for quality assurance	
Weekly	27 (55)
Monthly	13 (27)
Every few months	7 (14)
Quality assurance provider	
Project staff	37 (76)
Specialists	35 (71)
Non-specialists	16 (33)
Service users or carers	2 (4)

Over half of projects trained health workers, including specialist, non-specialist or lay health workers, to deliver mental health services (Fig. 1). Other providers trained to deliver mental health services included school staff and spiritual or traditional healers, with school staff being the third most commonly trained type of provider. Training targets were exceeded for all types of providers, except for lay health workers. However, even in this case almost 90% of the intended lay health workers were trained. For other provider types, projects exceeded their training targets by more than a third. For example, the number of specialists trained was twice as large as originally intended.

### Delivery (Outcomes 3–5)

**Fig. 2:**
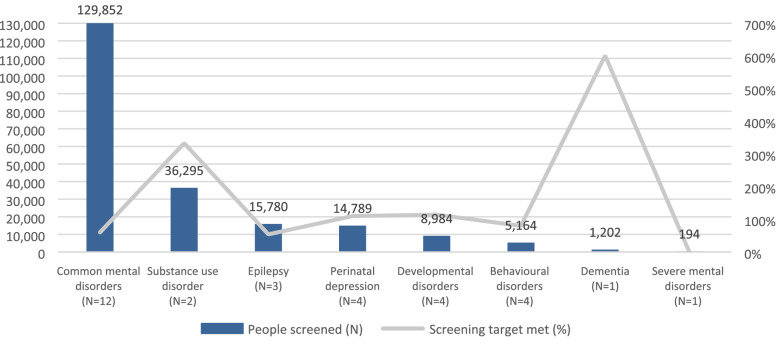
Number of people screened by MNS disorder. *Missing disaggregated data for 109,673 people who were screened by 10 projects

**Fig. 3:**
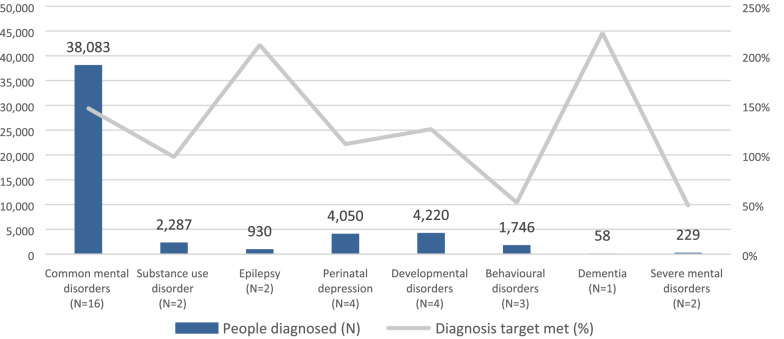
Number of people diagnosed by MNS disorder. * Missing disaggregated data for 27,655 people who were diagnosed by 14 projects

**Fig. 4:**
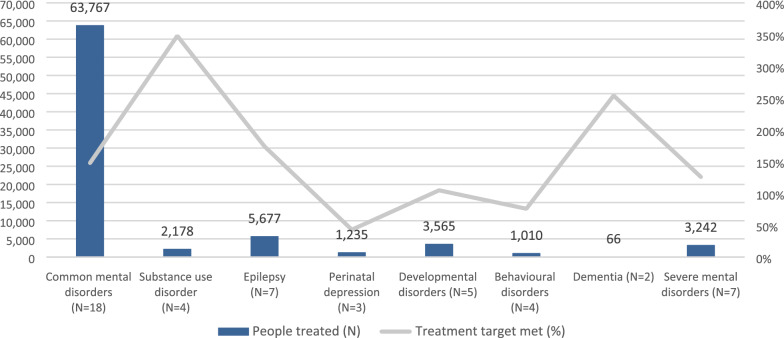
Number of people treated by MNS disorder. *Missing disaggregated data for 82,124 people who were treated by 18 projects

Services delivered included screening and diagnosis (n = 46), treatment (n = 49) and mental health promotion and awareness (n = 22). Table 4 presents the characteristics of the services delivered by all projects, and Figs. 2, 3 and 4 present the total number of people who were screened, diagnosed and who accessed treatment, as well as the extent to which targets set by projects were achieved.

**Table 4:** General characteristics of screening, treatment and promotion interventions delivered by GCC projects

Type of screening methods	GCC projects (n = 46) N (%)
Paper-based	31 (67)
mHealth	5 (11)
Paper-based and mHealth	10 (22)
Screening setting	
Community	21 (45)
Clinic	12 (27)
School	4 (8)
Multiple settings	9 (20)

Type of treatments delivered	GCC projects (n = 49) N (%)
Psychosocial interventions	35 (71)
Talk-based interventions	34 (69)
Pharmacological treatment	18 (37)
Setting of treatment delivery	
Community	37 (76)
Primary care clinics	29 (59)
Home	16 (33)
Specialist clinics	13 (26)
School	8 (16)
Workplace	4 (8)
Treatment provider	
Non-specialist health workers	33 (67)
Lay health workers	27 (55)
Specialist health workers	24 (49)
School staff	5 (10)
Spiritual or traditional healers	4 (8)

Type of promotional activities	GCC projects (n = 22) N (%)
Face-to-face activities	19 (86)
Distribution of printed materials	16 (73)
Media	9 (41)
Online/mHealth	3 (14)

Screenings were most commonly conducted at the community level (45%) using paper-based tools (67%). Five projects that conducted screenings did not report outcome data, however the remaining 41 projects reported screening a total of 321,933 people, primarily for common mental disorders. The three projects with the highest number of people screened used technological solutions for screening and reported screening between 30,000 to 45,000 people. Forty-five projects reported diagnosing 75,208 people, 51% of which were diagnosed with a common mental disorder.

Most treatment interventions provided by projects consisted of talk-based (69%) and psychosocial interventions (71%) delivered at the community level (76%) by non-specialist health workers (67%) or lay health workers (55%). All 49 projects that included a treatment intervention reported the number who accessed treatment. A total of 162,915 people received treatment, nearly half receiving treatment for common mental disorders.

Most projects focused on more than one disorder and exceeded all their targets for every disorder (Figs. 2, 3 and 4). Fewer people than intended were screened for common mental disorders and epilepsy (i.e. 60% and 54%, respectively), but many more were diagnosed (i.e. 211% and 147%, respectively) and treated (i.e. 174% and 148%, respectively) for these disorders than originally expected. All targets for substance use disorders, developmental disorders and dementia were exceeded, although it is worth noting that some of projects set very low targets for these disorders (e.g. dementia). In the case of perinatal depression, despite exceeding screening and diagnosis targets, projects only provided treatment to 43% of their intended target for this disorder.

## Discussion

This paper describes a diverse sample of mental health projects funded by GCC. Our findings highlight the utility of a ToC-driven process to define and map portfolio-level indicators across a pathway of change, to identify common outcomes, guide evaluation of challenges and drivers of successful implementation and identify knowledge gaps. These gaps were explored further using qualitative methods, the results of which are reported elsewhere in this series and referenced in our discussion below. Recommendations to funders and implementers involved in similar evaluation processes are summarised in Box 1.

### Capacity building and service delivery

Most projects exceeded their training targets for capacity-building. It is likely that unexpected staff turnover forced projects to train more service providers than initially planned. Retention emerged as a key barrier in the qualitative component of our research, particularly during the training and service delivery phases of project implementation [21]. Previous studies have reported high turnover among health workers to be a common and significant challenge to implementation, especially for time-limited projects [31]. The fact that lay health workers were the only type of provider for which training targets were not exceeded could perhaps be a reflection of comparatively low turnover in this cadre, as described by some interviewees who suggested that participation in the project offered valued opportunities for lay people to advance their careers in contexts of high unemployment [21].

More than 80% of projects included screening and treatment components, whereas only around 40% included mental health promotion and awareness-raising activities. Improvements in service delivery benefit the population in need of treatment, but further action on mental health promotion and awareness is important for the wider population at risk. In particular, investment in promotion and awareness is needed to strengthen early identification in young people and can be cost-effective, with potentially high returns [32]. However, activities that increase detection of mental disorders should be coupled with efforts to strengthen mental health systems, to avoid generating demand that cannot be safely and effectively met by existing services.

Over- or under-estimating the level of demand for services was a commonly faced barrier to successful delivery for grantees [23]. Reliance on isolated prevalence estimates [33], lack of appropriate epidemiological statistics on population mental health [34], limited understanding of the attitudes on help seeking for mental health [35], fragmented routine data around existing service use [36], and lack of validation of screening tools [37] could all be contributing factors. Screening targets for common mental disorders and epilepsy were the most likely to be underachieved but treatment and diagnosis targets for these disorders were more likely to be overachieved. Screening, diagnosis and treatment targets for substance use disorders, dementia and developmental disorders were overachieved and projects targeting perinatal depression overachieved screening and diagnosis targets but underachieved treatment targets.

We can take away from these differences in delivery outcomes across various MNS disorders three possible lessons. First is the need for different activities and interventions to engage and treat people with different MNS disorders. It is likely that a one-size-fits-all approach will leave certain groups underserved. Second is that there may be greater knowledge gaps for some disorders, making it more difficult to accurately estimate and plan for service delivery. Funders may need to make special considerations for projects targeting disorders that have been historically ignored; for example, by offering longer timelines and additional resources to carry out formative research before setting targets. Third is that the need for special consideration also extends to our own analysis. While we did disaggregate quantitative data on delivery outcomes by disorder, any conclusions we might seek to draw from this evaluation at the portfolio level will be heavily skewed toward common mental disorders, which were those most frequently targeted by GCC projects.

Bearing this final challenge in mind, it is promising to note that GCC’s investment has resulted in large numbers of human resources trained in mental health, of people screened for mental health conditions, and of people accessing care in LMICs. This offers hope for the possibility of scaling up mental health care in low-resource settings around the world. However, our analysis does not answer the all-important question of whether and how this care actually benefits the individuals, services and communities involved. Answering this more difficult question requires overcoming some of the limitations described below and in our recommendations for portfolio-level analysis [Box 1].

## Strengths and limitations

The use of a multi-method ToC approach was a strength of this research. ToC workshops can facilitate the involvement of multiple stakeholder groups in the mapping process [24]. In the case of GCC’s portfolio-level ToC, this process allowed for the identification of indicators at each step of a collective pathway of change—including indicators that were not already accounted for in GCC’s pre-existing project evaluation framework (RMAF). Data collected against these indicators allowed for the aggregation of output data—such as number of people diagnosed and treated—across projects, helping GCC to communicate the performance of its portfolio to key stakeholders [38]. The ToC mapping also helped to pinpoint where on the pathway grantees commonly experienced challenges, which were explored further through qualitative analysis (see companion papers in this series for detailed examples).

However, some components of the ToC pathway proved difficult to measure either quantitatively or through sufficiently standardised qualitative methods (e.g. summative content analysis) to enable aggregation. For example, during interviews both context and stakeholder engagement were identified as highly relevant to the process of implementation, and this important finding would have been missed if we had relied solely on quantitative data. Grantees described stakeholder engagement as one of the key factors determining the success or failure of their projects. Strong stakeholder relationships were built over time—sometimes long before applying to GCC—and could be an important output in and of themselves, with long-term implications for sustainability [22].

The heterogeneity of the sample presented challenges. While there were common elements across most projects, small subgroup sample sizes made it difficult to make meaningful comparisons. In addition, the heterogeneity of the sample contributed to large amounts of missing data (i.e. when a component was not applicable to a specific project). Anecdotal evidence from grantees suggests reporting fatigue was also a factor. Routine reporting on health projects can prove burdensome, particularly in the absence of adequate information systems [39]. We suspect this may have been aggravated by the large amount of Core Metrics data requested, as well as the frequency of reporting required.

Large amounts of missing data, particularly on outcomes related to effectiveness, meant that it was not possible to carry out a QCA investigating which factors were sufficient or necessary to achieve impact across the full GCC portfolio. However, qualifying in advance what success at each step on the pathway should look like would also have been problematic. The variation we observed among project targets set by grantees suggests that these were not well-defined from the outset, an issue explored further in Qureshi et al. [23]. Importantly, the extent to which targets were achieved did not necessarily reflect the degree to which implementation was successful. For example, projects with high staff turnover may have been forced to recruit and train new staff, exceeding their training targets while still facing human resource shortages—as described in Endale et al. [21].

Finally, it is important to keep in mind the potential for reporting bias. Core Metrics data were extracted from reports that grantees submitted to their funder. It is also possible that grantees may have knowingly under- or over-estimated targets, either setting expectations low to mitigate risk of underperformance, or perhaps overpromising to improve the value-for-money proposition of their projects at proposal stage. This analysis is also skewed toward those projects that were able to successfully report outcome data during the GCC-funded timeframe. Projects that either never communicated belated results or never completed their evaluations may be more likely to have experienced significant challenges in delivering on other expectations, as well—painting a more optimistic picture of the portfolio’s performance.

**Table:** 

Box 1. Recommendations for the application of Theory of Change to portfolio-level evaluation	
1. Engage grantees in designing reporting templates to ensure templates provide meaningful information and they do not cause reporting burden 2. Allocate resources for the development of relevant indicators for complex and difficult-to-capture components, e.g. context and stakeholder engagement 3. Allocate resources for the contextual validation of measurement tools, especially for less common disorders and settings where regional evidence is also lacking 4. Offer technical support to grantees to design and execute rigorous evaluations of their individual impact 5. Consider delays in obtaining final evaluation data when planning the timeline for portfolio-level evaluation 6. Use qualitative methods to complement and further explore quantitative findings, especially for complex and difficult-to-capture components	

## Conclusions

Despite growing interest by funders, there is little prior evidence or experience of ToC-driven evaluation of funding portfolios documented in either the academic or grey literature. The application of ToC to Grand Challenges Canada’s global mental health funding portfolio and the resulting Core Metrics framework offered an important opportunity to examine common aspects of diverse projects, which when coupled with qualitative exploration of complex themes and project-specific issues, helped to harness key learning from one of the largest investments in global mental health to-date. Future efforts to produce more definitive evaluations of global mental health funding portfolios should focus on supporting grantees to thoroughly monitor and evaluate their projects through to completion, recognising that the same challenges encountered in implementing mental health projects in low-resource settings are likely to also affect the quality and completeness of the data they generate. Supporting grantees to overcome these challenges will not only help funders to deliver impact through their funding portfolios, but also to measure their progress along the way.

## Data Availability

The quantitative data that support the findings of this study are available from GCC, but restrictions apply to the availability of these data, which were used under license for the current study, and so are not publicly available. These data are however available from the authors upon reasonable request and with permission of GCC. The qualitative data generated during the current study are not publicly available due to the sensitivity of discussions surrounding the performance of grantees’ projects but are available from the corresponding author upon reasonable request.

## Appendix 1

See Fig. 5.

## Abbreviations

AIDS: Acquired immunodeficiency syndrome
CAD: Canadian dollars
CQI: Continuous quality improvement
GCC: Grand Challenges Canada
HIV: Human immunodeficiency virus
IBM: International Business Machines Corporation
LMIC: Low- or middle-income country
LSHTM: London School of Hygiene and Tropical Medicine
MHIN: Mental Health Innovation Network
MNS: Mental, neurological and substance use
PRIME: PRogramme for Improving Mental hEalth care
PTSD: Post-traumatic stress disorder
QCA: Qualitative comparative analysis
RMAF: Results-based Management and Accountability Framework
SPSS: Statistical Package for the Social Sciences
ToC: Theory of Change
USD: United States dollars
WHO: World Health Organization
